# On Dr. Brenan's Terebinthinate Remedy in Puerperal Fever

**Published:** 1815-03

**Authors:** Robert Kinglake

**Affiliations:** Taunton


*For the Medical and Physical Journal.*

*On Dr. Brenan's Terebinthinate Remedy in Puerperal Fever;* by Dr. Kinglake.

THE practical reports lately given to the medical world by Dr. Brenan on the astonishing efficacy of spirit of turpentine in the cure of puerperal fever, deserve close and critical attention.* No description of disease is, perhaps, so insidious in its approach, so deceptious in its progress, and

---

* See Med. and Phys. Journal for Nov. 1814, p. 403.

so unexpectedly mortal, as puerperal affections. After the alarms and difficulties attending parturition have subsided, and the most happy assurances are apparently afforded of a favourable issue, pains of no threatening violence in the uterine region, extending to the general cavity of the abdomen, announce the occurrence of a very formidable complaint, which, if not speedily repressed and overcome, but too commonly proves destructive.

That the character of the disease is at the onset inflammatory, and that in this form, if not timely checked, it lays the ground of irrecoverable mischief, is, it may be presumed, undeniable; but it is obvious, that, in a disease forming and developing itself under so much disguise and ambiguity as is known to be the case in puerperal affection, no opportunity is afforded for snatching the menaced victim from the impending danger. The mortal stage is often unhappily attained before effectual aid could have been applied, and, under such circumstances, the patient is unexpectedly cut off.

Whether this disease be induced by a morbid irritability of the uterine system, propagating itself to the peritoneal covering of the contents of the abdomen, or from mismanagement either before, at the time, or after the period of parturition, the character and consequence of the disease would be in principle the same. Inordinate action, bordering on, or actually amounting to, a state of inflammation, constitutes the uniform and dangerous nature of the disease. Its seat being in the membranous structure, occasions it to assume more of the erysipelatous than phlegmonous kind of inflammatory excitement; hence, the comparative slightness of pain, and the rapidity with which it becomes indefinitely diffused over the membranous structure of the abdominal cavity. Dangerous and untractable as this disease is when suffered to proceed, it may in its early stage be generally restrained and overcome by copious bleeding and extensive blistering over the region of the abdomen. This mode of remedy is the most efficient in subduing inflammatory affections of the cavities, and evinces, from its commonly availing in puerperal affection, that the disease is essentially inflammatory. But, when the complaint has proceeded long enough to have exhausted the vital power of the affected vessels to a degree but little short of the state of death, gangrene must be near at hand, and, indeed, most commonly occurs, bringing with it inevitable destruction.

This seems to be the condition of disease in which Dr. Brenan has successfully employed Spirit of Turpentine. It will not be contended that a part actually dead can be restored to life, but it is possible that the dying state may be prevented prevented from reaching its mortal termination; and the question is, whether the high stimulus of turpentine is peculiarly equal to this wonderful effect. Two opinions cannot be held on the fact, that, in the greater number of Dr. Brenan's reports of the efficacy of his remedy, death must necessarily have been the result, but for the extraordinary efficacy of his terebinthinate remedy.

The salutary influence of turpentine in the griping affection of horses, is immediate and most effectual. Within ten minutes after taking an adequate dose of this article, the animal will stale copiously, and be completely relieved. Large quantities of turpentine may be taken with impunity. In a case of tænia, it was given under my direction to the extent of six drams a day during several weeks, and without any other inconvenience than that of passing an unnatural quantity of urine, and the insensible perspiration becoming strongly impregnated with its peculiar odour. No material advantage arose from it in dislodging the supposed tænia. It has been not only the established practice from remote antiquity down to the present time, but is the obvious suggestion of common sense, to endeavour to sustain the sinking state from exhaustion, however the loss of power may have been induced, whether by excessive or deficient excitement. The close of inflammatory diseases is, therefore, treated with stimulant remedies; but they are in general resorted to after gangrene has actually taken place, and death has consequently become inevitable. The peculiarly active and diffusible powers of turpentine, may, perhaps, render it a more effectual stimulant in cases verging on gangrene, such as are apt to present in puerperal disease, than any other that could be employed. Should this be the case, it will be entitled to decided preference. The extent to which it has been given by Dr. Brenan, is probably the main circumstance of its efficacy. In limited and fearful doses, it would, probably, achieve nothing, whilst in *half-ounce* doses, it might prove salutarily efficient. It will be vindicable to try what may be accomplished by it in those cases in which it is affirmed to have succeeded, and in which no known mode of remedy would be likely to avail.

How far it would be adviseable to employ the remedy at an early stage of the disease, unpreceded by bleeding, blistering, and intestinal discharges, is a subject for grave consideration. It would appear to me, that in that active stage of the disease it would be much less likely to prove salutary than in the more advanced progress, when the enfeebled remains of life might have nearly reached the point of mortal exhaustion. Should this remedy, on adequate trial, be found to be effectual tual in the sinking stage of puerperal affection, the pathological analogies which the fact is calculated to unfold, may be made available to most important objects in the general treatment of disease. The practice of medicine is yet lamentably imperfect. Boasted remedies are on trial ascertained to be very defective, and some of the accredited principles by which they are authorised and kept in vogue, will, perhaps, be found not to be unassailably correct.

*Taunton;* ROBERT KINGLAKE.

